# A Comprehensive Analysis of the Impact of Inorganic Matter on Membrane Organic Fouling: A Mini Review

**DOI:** 10.3390/membranes13100837

**Published:** 2023-10-20

**Authors:** Qiusheng Gao, Liang Duan, Yanyan Jia, Hengliang Zhang, Jianing Liu, Wei Yang

**Affiliations:** 1 State Key Laboratory of Environmental Criteria and Risk Assessment, Chinese Research Academy of Environmental Sciences, Beijing 100012, China; 201931470030@mail.bnu.edu.cn (Q.G.); yy_jia@tongji.edu.cn (Y.J.); 201831470030@mail.bnu.edu.cn (H.Z.); liujianing20@mails.ucas.ac.cn (J.L.); 2 Institute of Water Ecology and Environment, Chinese Research Academy of Environmental Sciences, Beijing 100012, China; 3 College of Water Sciences, Beijing Normal University, Beijing 100875, China; 4 Institute of Ecology, Chinese Research Academy of Environmental Sciences, Beijing 100012, China

**Keywords:** inorganic matter, organic matter, membrane fouling, interactions

## Abstract

Membrane fouling is a non-negligible issue affecting the performance of membrane systems. Particularly, organic fouling is the most persistent and severe form of fouling. The complexation between inorganic and organic matter may exacerbate membrane organic fouling. This mini review systematically analyzes the role of inorganic matter in membrane organic fouling. Inorganic substances, such as metal ions and silica, can interact with organic foulants like humic acids, polysaccharides, and proteins through ionic bonding, hydrogen bonding, coordination, and van der Waals interactions. These interactions facilitate the formation of larger aggregates that exacerbate fouling, especially for reverse osmosis membranes. Molecular simulations using molecular dynamics (MD) and density functional theory (DFT) provide valuable mechanistic insights complementing fouling experiments. Polysaccharide fouling is mainly governed by transparent exopolymer particle (TEP) formations induced by inorganic ion bridging. Inorganic coagulants like aluminum and iron salts mitigate fouling for ultrafiltration but not reverse osmosis membranes. This review summarizes the effects of critical inorganic constituents on fouling by major organic foulants, providing an important reference for membrane fouling modeling and fouling control strategies.

## 1. Introduction

The rapid development of human society has triggered a population explosion, which has brought about massive water pollution. The problem of water scarcity has gradually come to the forefront. Membrane treatment is considered a promising technology widely used in water reclamation, such as deep purification of secondary effluent from wastewater treatment plants, seawater desalination, and drinking water purification [1,2,3,4,5]. Its advantages include its simple operation, being energy saving, and having high efficiency and high safety [6]. Membrane treatments commonly employed in water purification include forward osmosis (FO), microfiltration (MF), ultrafiltration (UF), nanofiltration (NF), and reverse osmosis (RO). Among these, only FO does not require the application of external pressure to achieve separation. The process is driven by the osmotic pressure difference created between the feed solution and draw solution [7,8,9]. The remaining types of filtration technology are driven by operation pressure, which accelerates membrane fouling.

Membrane fouling is a complex process that occurs as a result of the interaction between pollutants in the feed water and the membrane surface, leading to deposition and adsorption on the membrane. It is an inevitable process that can gradually reduce the permeate flux during membrane filtration, resulting in increased energy consumption and a shortened membrane service life [10,11].

**Figure 1:**
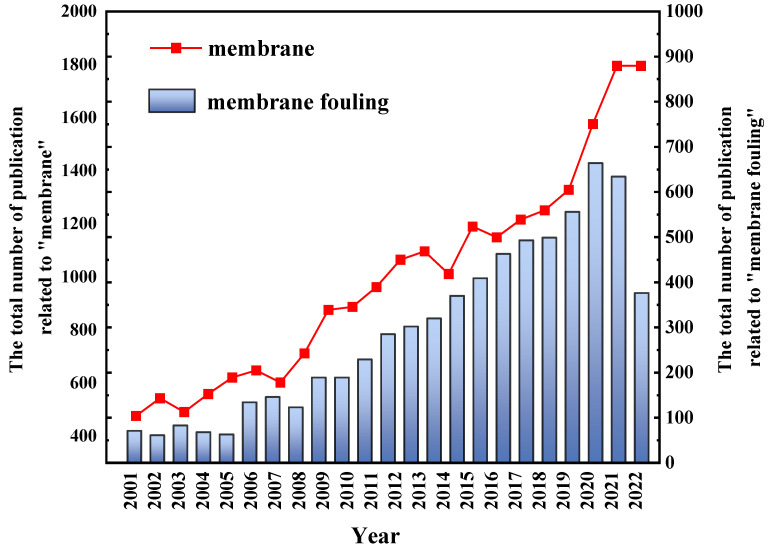
The total number of publications related to “membrane” and “membrane fouling”.

Based on the number of articles published on membranes and membrane fouling since 2001 (Figure 1), membrane technology has received increasing attention, and research on membrane fouling accounts for almost half of this research. Membrane fouling is evidently a non-negligible issue in membrane research.

Membrane fouling includes inorganic fouling, organic fouling, colloidal fouling, and biofouling. Among these, organic fouling and biofouling are the most severe types of fouling [12]. Compared with biofouling, organic fouling occurs throughout the entire membrane separation process and is difficult to control. Previous studies have demonstrated that using organic matter alone minimizes membrane fouling. Conversely, the introduction of inorganic matter, particularly metal ions including Ca^2+^ [13], Mg^2+^ [12], Al^3+^ [14], and Mn^2+^ [15], can significantly exacerbate membrane fouling by complexing with the organic matter [16]. Even when the concentration of inorganic species is as low as 10^−5^–10^−3^ mol/L, the flux can show significant declines. Moreover, in practical applications of membrane technology, organic matter generally coexists with inorganic matter in the feed water. Therefore, studying the impact of inorganic matter on membrane organic fouling is important.

Three fouling control strategies are membrane modification, regular membrane cleaning, and pretreatment technologies. However, the prerequisite for the above strategies is understanding the mechanism of membrane fouling. From about 300 annual publications in 2011 to nearly 1000 today, more than 6000 articles on membrane fouling have laid a strong foundation for the elucidation of membrane fouling mechanisms under various conditions.

**Figure 2:**
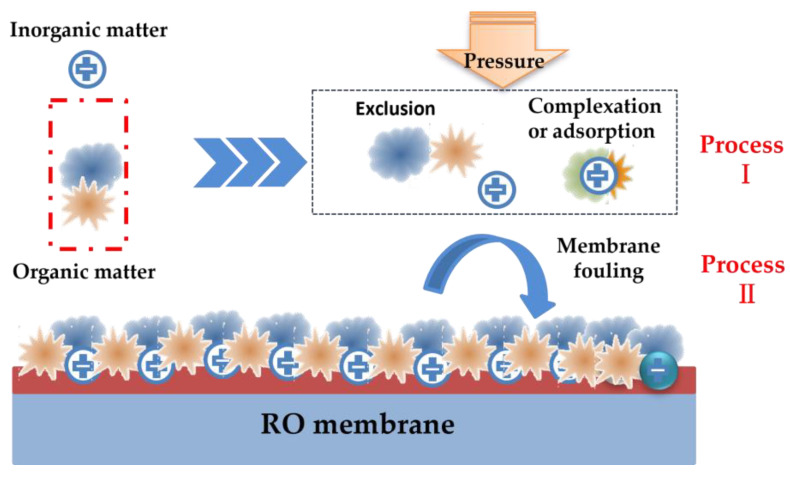
Schematic diagram of membrane organic fouling processes.

There have been many reviews on membrane fouling, mainly focusing on types of membrane fouling and control strategies [10,17,18,19], membrane fouling caused by certain kinds of pollutants [20,21,22,23], thermodynamic mechanisms [24], and mathematical modeling [25,26,27]. However, there are few reviews on the effects of inorganic substances on membrane organic fouling. The essence of membrane organic fouling is that organic and inorganic substances in the feed water interact to form aggregates deposited and adsorbed on the membrane, reducing membrane permeability. This can be divided into two processes: the pollutant interaction process and membrane fouling process (Figure 2). Firstly, organic and inorganic substances in the feed water interact with each other according to their own physical and chemical properties, such as complexation, adsorption, or mutual exclusion. Then, based on the results of the above interactions, the composite pollutants accumulate on the membrane to form substantial membrane fouling. These processes are worth summarizing systematically. 

Therefore, this study systematically analyzes the reaction processes in membrane treatment systems with inorganic and organic substances, focusing on the role of functional groups and chemical bonds during the reaction processes, and the complementary role of molecular (density functional theory (DFT) and molecular dynamics (MD)) simulations in explaining the fouling mechanism during membrane fouling experiments. Then, the fouling behavior of various inorganic–organic complexes on the membrane was summarized, and the role of key inorganic substances was identified. This article provides a comprehensive summary of the effects of inorganic substances that have been reported in membrane fouling research. It can serve as an important reference for membrane fouling modeling and fouling control strategies.

## 2. Interactions between Inorganic and Organic Matter

Studies have shown that natural organic matter (NOM) is rich in water environments, and is the main culprit of membrane fouling [28]; humic acid, polysaccharides, and protein are the key components of NOM [29]. In studies of organic fouling, these three components are usually used to represent NOM, so for this article, we reviewed the effect of inorganic species on membrane organic fouling caused by humic acid (HA), polysaccharides, and protein.

### 2.1. Humic Acid (HA)

Among the components of NOM, HA is a group of organic polyelectrolytes metabolized from natural and biological degradation. It occupies up to 80% of the total organic carbon in natural waters; it is abundant in surface water, seawater, groundwater, wastewater, and landfill leachate [30,31], with concentrations ranging from a few mg/L to a few hundred mg/L [32]. HA contains three primary functional groups: carboxylic acids (COOH), phenolic alcohols (OH), and methoxy carbonyls (C=O) [33]. These functional groups can interact with inorganic matter via electrical double layers, neutralization, or complexation, and these interactions significantly aggravate membrane fouling. At low silica particle concentrations, silica adsorbs onto HA aggregates through hydrogen bonds, bridging the HA network together to form large foulants due to the silica–silica interactions above the critical silica concentration [16]. Similar to silica, Ca^2+^ and Mg^2+^ interact with the functional groups of HA through hydrophobic interactions, bridging the HA molecules together to form aggregates that cause membrane fouling. Due to its stronger electrical neutralization capacity and binding force with HA compared with Mg^2+^, Ca^2+^ forms more aggregates when complexed with HA, leading to increased membrane fouling [31]. The preserved humic acid components after UF filtration (PHACUF) exhibit a higher affinity for Mg^2+^ due to the presence of more than 81.1% hydrophobic or weakly hydrophobic fractions in the HA after ultrafiltration. These fractions possess a higher charge density and a greater proportion of aliphatic compounds compared with the original HA. Moreover, the smaller ionic radius and significantly higher charge density of Mg^2+^ allow for better specific charge and complexing ability than Ca^2+^, enabling easier interaction with the carboxyl group of PHACUF and resulting in stronger electrostatic adsorption [34].

In summary, the interaction between inorganic substances and humic acids can affect the structure, properties, and behavior of humic acids, mainly in the following two roles:

1. Ligand interaction: Some inorganic substances can form ligand bonds with functional groups (e.g., carboxylic acids, hydroxy acids, phenols, etc.) in humic acids. This coordination can change the structure of humic acids, affecting their solubility, charge density, and chemical reactivity.
2. Adsorption: Inorganic substances can be adsorbed on the surface of humic acid molecules to form an adsorption layer. This adsorption can affect the surface properties, adsorption capacity, and environmental behavior of humic acids.

### 2.2. Polysaccharides

Polysaccharides are a type of organic matter that occupies a large proportion of soluble microbial products (SMP) and extracellular polymeric substances (EPS) [35,36]. Due to its abundance and gelling properties, polysaccharide fouling plays a very important role in organic membrane fouling and has been widely reported in membrane filtration studies [37,38,39]. In its early stages, research on membrane fouling caused by polysaccharides mainly focused on the type and concentration of polysaccharides and calcium and magnesium inorganic ions. However, advancements in fouling research have revealed that transparent exopolymer particles (TEP), which arise from the crosslinking of polysaccharides, are primarily responsible for membrane fouling in the membrane filtration process [12,13,40,41]. TEP are a kind of granular polysaccharide with a three-dimensional network structure; it has been widely reported that TEP have significant abundance in seawater, surface water, and wastewater [42,43]. Polysaccharides have a propensity to bind together, creating a substantial three-dimensional network structure, particularly when inorganic ions act as bridging agents. This network can become even more extensive. Therefore, TEP can serve as an indicator of the degree of crosslinking in polysaccharides. Furthermore, the concentration and size of TEP are crucial factors when explaining membrane fouling.

**Figure 3:**
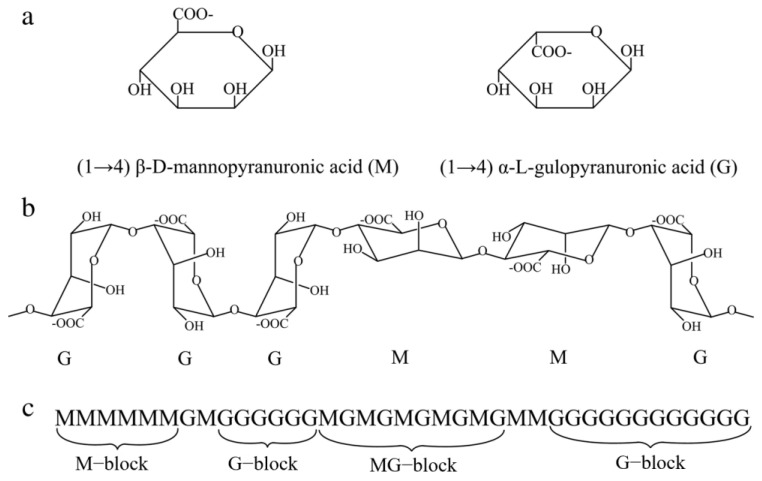
Structural characteristics of the alginate molecule: (**a**) an alginate monomer; (**b**) alginate chain conformation; (**c**) alginate block distribution [44].

In studies of membrane fouling caused by polysaccharides, sodium alginate (SA) is usually selected to represent polysaccharide-like pollutants. SA (Figure 3) is a linear binary copolymer containing (1,4)-linked β-d-mannuronate (M) and αl-guluronate (G) residues, which are randomly arranged along the chain into consecutive M residues (MM-blocks), consecutive G residues (GGblocks), and alternative M and G residues (MG-blocks) [44]. Each residue has plenty of carboxylate groups and hydroxyl groups, providing abundant reaction sites for bridging via inorganic ions.

The roles of Ca^2+^ and Mg^2+^ on alginate fouling have been well studied; they both promote interaction between alginate molecules, and the resulting bridging promotes the formation of TEP. However, these two ions bind differently to alginate. Ca^2+^ can form ionic bonds with the carboxylate groups (COO−) and hydrogen bonds with the hydroxyl group or the oxygen of the ether bond [45]. On the other hand, Mg^2+^ has large and rigid hydration shells, and the repulsive interactions between hydrated Mg^2+^ reduce the local concentration of Mg^2+^, the charge density, and the electric double layer field of the alginate chain. Thus, the binding affinity of Mg^2+^ to alginate is much weaker than Ca^2+^. For Mg^2+^ binding, ionic bonds are not possible, especially at the low concentrations of polysaccharides in membrane systems. In addition, the hydrated Mg^2+^ cannot lose all the water molecules in its hydration shells. Therefore, hydrogen bonds can form between the Mg^2+^ ion and the oxygen atoms of the COO− (Mg^2+^⋯O(COO−)) and OH−(Mg^2+^⋯O(OH)) groups in the alginate chains [12]. In particular, the hydrated Mg^2+^ ion may share water molecules in its first hydration shell with the COO− groups of alginates. These various binding patterns with polysaccharides result in differences in TEP formation: at constant alginate concentrations, TEP concentrations first increased and then decreased with increasing Ca^2+^ concentration, whereas it increased with increasing Mg^2+^ concentration. However, the patterns of change in the size of TEP are the same, that is, they all become larger as the concentration of Ca^2+^ and Mg^2+^ increases [45]. What is more, although MG−, MM−, and GG-blocks have the same functional groups, their binding ability to Ca^2+^ and Mg^2+^ differed, with GG-blocks > MM-blocks > MG-blocks, and was dependent on the molecule chains [12].

In addition to detailed studies of Ca^2+^ and Mg^2+^, the roles of Al^3+^-SA and silicon-SA interactions during the membrane filtration process have also been reported [14,46]. Similar to Ca^2+^ and Mg^2+^, Al^3+^ coordinates with the carboxyl group to form TEP. The binding sites of silica are the carboxyl and hydroxyl groups. 

There are many kinds of polysaccharides, and the structures of different polysaccharides also vary widely. It is not sufficient to examine only alginate as a representative of polysaccharides to conduct relevant studies, but there are presently only a few studies exploring the combined effects of other polysaccharides and inorganic matters. Tong et al. [38] investigated the interactions between polysaccharides with different structures and Ca^2+^. Straight-chain polysaccharides with carboxyl groups (alginate and gellan gum) tended to bind to Ca^2+^ and accelerate the formation of fouling. The effect of Ca^2+^ on a branched polysaccharide with a carboxyl group (xanthan gum) was not obvious, whereas the fouling behavior of a straight polysaccharide without a carboxyl group (guar gum) was similar to that without Ca^2+^. In addition, other studies [47] have investigated the interaction between the neutral polysaccharide dextran and Ca^2+^, and found that Ca^2+^ had a certain inhibitory effect on fouling, although this effect was weak. This is because dextran is neutral, so it is difficult for it to have an effect through electrostatic effects. 

In summary, there are multiple types of interactions between inorganic substances and polysaccharides, and these interactions can affect the structure, function, and properties of polysaccharides, most intuitively expressed by TEP. These interactions fall into the following four categories:

1. Ion-exchange interactions: Inorganic ions can undergo an ion exchange with charged groups (e.g., carboxyl groups, hydroxyl groups, etc.) in polysaccharides. This can change the charge state of the polysaccharide, affecting the ionic balance of the solution and the colloidal properties of the polysaccharide.
2. Hydrogen bonding: Inorganic substances can form hydrogen bonds with hydrogen bonding acceptors (e.g., hydroxyl, amine groups, etc.) in polysaccharides. This hydrogen bonding can stabilize the structure of polysaccharides and affect their solubility, viscosity, and gel-forming ability.
3. Coordination: Some inorganic ions can form coordination bonds with functional groups (e.g., hydroxyl, amine groups, etc.) in polysaccharides. This coordination can affect the structure and stability of polysaccharides, but also change their optical properties and catalytic activity.
4. Van der Waals force interactions: There are also van der Waals force interactions between inorganic substances and polysaccharides, such as intermolecular attraction, electrostatic interactions, etc., which can affect their mutual adsorption and cohesion properties.

### 2.3. Protein

Proteins are common macromolecular organic substances that are similar to polysaccharides and are major components of SMP and EPS. As with HA and polysaccharides, they were thought to be one of the main foulants of the membrane during the membrane filtration process.

**Figure 4:**
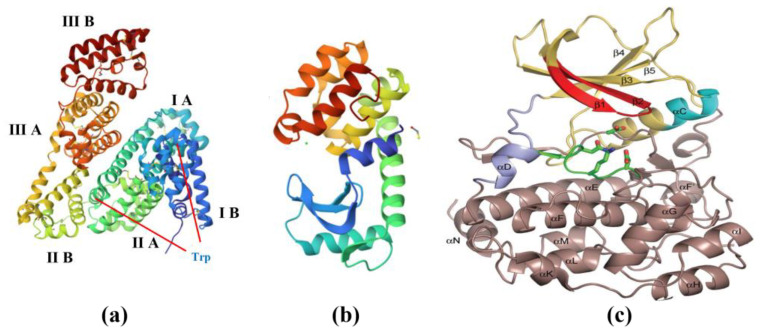
Molecular structure of proteins: (**a**) bovine serum albumin; (**b**) lysozyme; (**c**) casein. https://www.rcsb.org (accessed on 28 August 2023).

In studies of membrane fouling caused by proteins, bovine serum albumin (BSA) is always chosen to represent protein-like pollutants. The BSA molecule is elliptical (14 × 4 × 4 nm) with a negative charge, is endogenously fluorescent, and has an isoelectric point and molecular weight of approximately 4.7 (pH) and 66 KDa, respectively [48]. As shown in Figure 4a, three homology regions (regions I, II, and III) comprise the three-dimensional structure of BSA, each consisting of a macrocycle–microcycle–macrocycle triad. Each BSA molecule contains two tryptophan (Trp) residues, where Trp-212 is located within the hydrophobic binding region of the protein and Trp-134 is located on the surface of the BSA molecule. When there is only BSA in the feed water, membrane fouling is mainly caused by protein aggregation from the cross-linking of BSA intermolecular disulfide bonds, especially at the isoelectric point [49]. However, membrane fouling could be more complex when inorganic matter coexists with BSA in the feed water. Ca^2+^ can form an ionic bridge between two neighboring carboxyl groups of different peptide chains, resulting in greater aggregation between BSA molecules, and thus more severe membrane fouling. In contrast, the bridging effect is weaker and membrane fouling is less severe for Mg^2+^. This phenomenon is mainly because Mg^2+^ has a higher charge density than Ca^2+^ and exhibits a greater salting-out effect [50,51]. The same bridging effect was also reported for Fe^3+^ and BSA [52]. The average adhesion force between silica and BSA is much higher than the average adhesion force between BSA molecules, which causes a large number of BSA molecules to adsorb on the surface of silica and form large aggregates [53]. This leads to the destabilization of BSA in solution and to the salting-out effect, causing the SI–BSA complexes to accumulate on the membrane surface.

Lysozymes (LYZ) (Figure 4b) are also a kind of protein found in various bodily fluids and tissues, such as tears, saliva, mucus, and egg whites. It is composed of 129 amino acids, with an isoelectric point and molecular weight of approximately 10.4 (pH) and 14.3 kDa, respectively [54]. Similar to BSA, LYZ aggregates with silica, but to a lesser extent than BSA because of its relatively lower molecular weight, lower concentration polarization modulus, and greater stability [53].

Casein (Figure 4c) is a complex protein found in milk, mainly consisting of four different types of protein molecules: αS1-casein, αS2-casein, β-casein, and κ-casein. The structure of casein can be described as long chains of amino acids that fold and interact with each other in various ways. The interaction between casein molecules and Ca^2+^ is mainly through the formation of Ca^2+^ bridges between casein molecules, forming larger aggregates of casein molecules [53].

In summary, there are a variety of interactions that can occur between inorganic substances and proteins that have important implications for protein structure, function, and stability. These interactions fall into the following three categories:

1. Ionic interactions: Inorganic substances can have ionic interactions, such as salt-bridging, with charged amino acid residues in proteins. This interaction can affect the secondary structure and stability of proteins.
2. Hydrogen bonding: Inorganic substances can have hydrogen bonding interactions with amino acid residues in proteins, including the carbonyl, hydroxyl, and amino groups of amino acids. The formation of hydrogen bonds can affect the folding and stability of proteins.
3. Non-covalent interactions: Inorganic substances can also interact with proteins through non-covalent interactions such as van der Waals forces, hydrophobic effects, and hydrophobic interactions. These interactions can affect the structure, folding, and interactions of proteins.

### 2.4. Molecular Simulation

Molecular simulation is a bridge between experiment and theory, and with the development of computing power and simulation theory, molecular simulation technology has been widely used in the environmental field. Recently, in the research of membrane contamination, some scholars have used density functional theory (DFT) or molecular dynamics (MD) simulations to further verify the membrane fouling mechanism and have achieved promising results [14,16,55,56,57].

#### 2.4.1. MD

MD simulation is a computational technique used to model and study the behavior and dynamics of atoms and molecules over time. In MD simulation, the positions and velocities of atoms or molecules are simulated based on classical mechanics. The interactions between atoms or molecules are described by a force field potential, which includes terms representing bond stretching, angle bending, and non-bonded interactions such as electrostatic forces and van der Waals forces. It provides valuable insights into the behavior and properties of molecular systems that are difficult or impossible to study experimentally, offering a powerful tool for scientific research and discovery [58,59,60,61]. 

**Figure 5:**
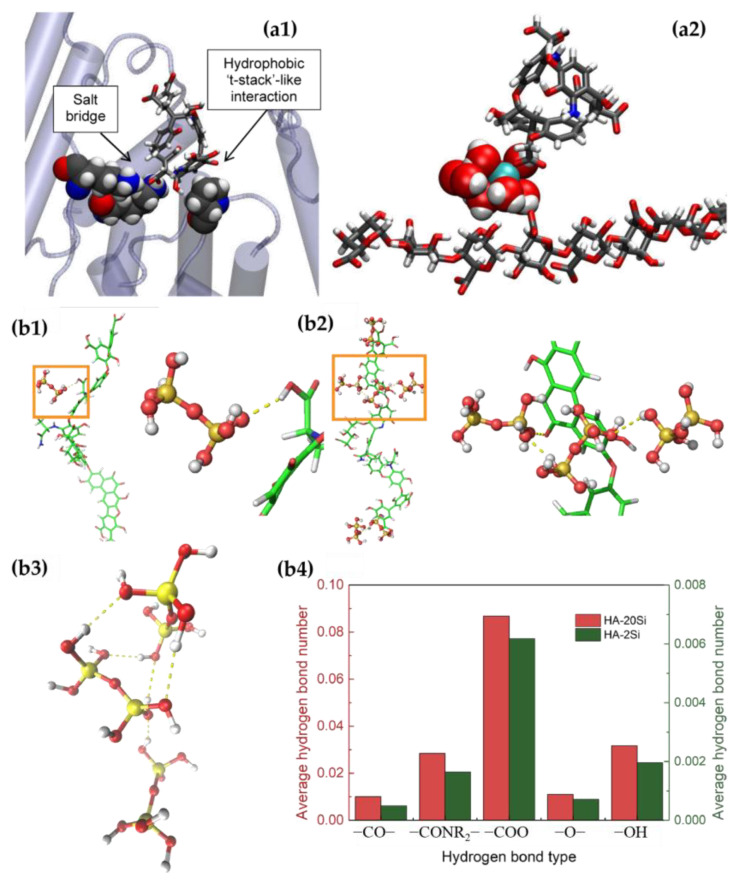
Molecular dynamics simulations: (**a1**) BSA − HA; (**a2**) SA − HA (N—blue, O—red, C —black, H—white, and Ca^2+^—green); (**b1**) a hydrogen bond formed in Case 1 between one Si molecule and one HA molecule; (**b2**) multiple hydrogen bonds formed in Case 2 between three Si molecules and one HA molecule; (**b3**) hydrogen bonds formed among different Si molecules in Case 2; (**b4**) the average hydrogen bond number formed by each functional group on the HA during the entire simulation (100 ns) [16,55].

With the help of MD, the process of SA and BSA respectively forming aggregates with HA was investigated in depth [55]. Our results showed that (Figure 5) electrostatic, hydrophobic, and hydrogen bonding were the main types of interactions in the BSA–HA system, and Ca^2+^ did not play a role. However, in the SA–HA system, the main interaction was water-mediated Ca^2+^ bridging between the deprotonated carboxylate portion of the humic acid molecule and the binding pocket on the alginate. The complexation between silica and HA was also further clarified by MD, revealing that hydrogen bonding was the main interaction force between HA and silica and occurred in two cases. In Case 1, one HA molecule interacts with two Si molecules, and in Case 2, one HA molecule interacts with twenty Si molecules. Silica can bind to hydroxyl (−OH), ether (−O−), carboxyl (−COOH), amide (−CONR_2_), and carbonyl (−CO−) groups on the HA main chain. However, the carboxyl group is the main functional group that binds to silica [16].

#### 2.4.2. DFT

DFT is a computational quantum mechanical method used to calculate the electronic properties of atoms, molecules, and solids. It is based on the concept of electron density, rather than wavefunctions, to describe the electronic structure. It provides valuable insight into molecular and solid-state systems, enabling predictions of properties such as molecular geometries, electronic energies, charge distributions, and reaction energies. It is widely used in chemistry, physics, and materials science, as it offers a good balance between accuracy and computational efficiency [62,63,64,65,66].

**Figure 6:**
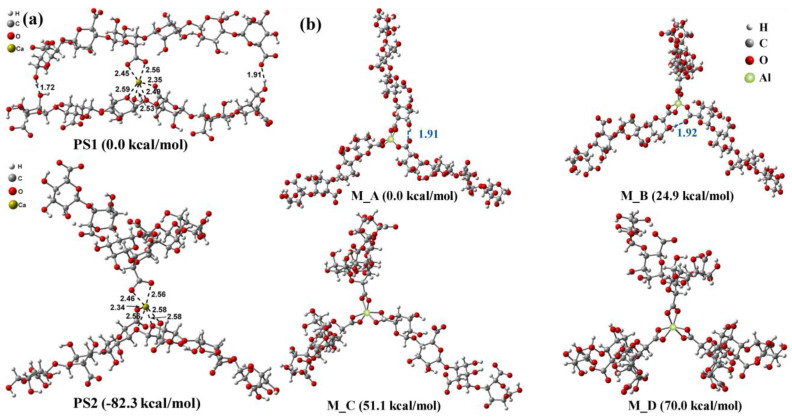
The potential geometrical configurations of the (**a**) calcium cation−alginate chain and (**b**) aluminum cation−alginate chain coordination compounds, and their calculated relative energies by DFT [14,55].

The mechanism of synergistic fouling by Ca^2+^ and SA was clarified by DFT calculations (Figure 6). The intramolecular ligand interactions between Ca^2+^ and SA chain appeared to be impossible, whereas Ca^2+^ could form six coordination bonds with oxygen-containing groups such as carboxyl, hydroxyl, and oxygen atoms of the sugar ring and glycosidic bond of SA. The two SA chains occurred preferentially through the intermolecular binding of Ca^2+^ to form a three-dimensional structure [57]. Similarly, the effect of PACl as a coagulant on the membrane fouling by SA during membrane treatment was calculated using DFT. The low concentration of Al^3+^ preferentially coordinated with the terminal carboxyl groups in the SA chains to form a homogeneous gel with low permeability. Higher concentrations of Al^3+^ would coordinate with non-terminal carboxyl groups and lower the negatively charged chains of alginate, resulting in a morphology shift/cake layer from gel layer to floc [14].

## 3. Membrane Fouling Behavior Based on Inorganic–Organic Interactions

Membrane fouling is a complex process caused by the deposition and adsorption of substances with different physical and chemical properties, as well as by mechanical, physical, or chemical interactions with the membrane surface during filtration [10]. As summarized above, inorganic matter in the feed water is bridged with various functional groups such as carboxyl, hydroxyl, and carbonyl groups in the organic matter to form reticulated or three-dimensional aggregates. Under operating pressure, these aggregates can cover the membrane surface and cause membrane fouling and a subsequent decrease in permeate flux. This process is called a pollutant–membrane interaction, which is closely related to the characteristics of the membrane itself and the physicochemical properties of the aggregates. Table 1 summarizes the patterns regarding the effects of inorganic and organic matter interactions on membrane fouling in recent years (nanofiltration is categorized as RO in this table). The level of fouling is expressed under the “Variation of Normalized Flux Compared with Organic Matter Fouling Only” heading, based on inorganic–organic interactions. Positive values mean that the addition of inorganic matter enhances the permeate flux and alleviates the membrane pollution, whereas negative values mean that the addition of inorganic matter reduces the water flux and increases the membrane pollution. A change in water flux with the increase in inorganic concentrations can also reflect an effect on membrane fouling.

The fouling behaviors of UF and RO are slightly different. As long as inorganic substances are present in the feed water, RO membrane fouling is exacerbated, and the degree of membrane fouling is directly proportional to the concentration of inorganic matter. Due to the small pore size (below 1 nm) of the RO membrane [67] and the high operating pressure, the pollutant is constantly compacted on the membrane, resulting in increasingly poor water permeability. However, in the UF system, when the feed water is injected with aluminum salts as coagulants, the membrane fouling is accidentally mitigated (the variation of normalized flux is positive), and the permeate flux is higher than when the organics existed alone. This is mainly because, although Al^3+^ can complex with organics to form an agglomerate, the agglomerate is deposited on the surface of the UF membrane in a loose and porous manner, with better adsorption, so that the organic contaminants are less compacted in the membrane pores and the removal rate of the organics by UF is also enhanced [68,69]. Similarly, using iron salts as a type of coagulant can also reduce UF membrane fouling and, unlike aluminum salts, it has a weaker effect and does not immediately reduce membrane fouling (the variation of normalized flux is positive). Instead, permeate flux gradually recovers as the Fe^3+^ concentration increases. This may due to iron salts producing a denser layer of contamination compared with aluminum salts [70]. Ca^2+^ has also been found to mitigate UF membrane fouling in the HA–Ca^2+^ system [71]. When the concentration of Ca^2+^ was low (1 mM), the addition of Ca^2+^ reduced the electrostatic repulsion between HA and UF membranes and promoted the formation of a dense fouling layer of HA on the membrane surface. With an increase in Ca^2+^ concentration, HA formed larger porous aggregates, whereas the hydration repulsion force was enhanced, weakening the adhesion between the pollutant and UF membrane. This reduced the membrane fouling, and recovered the permeate flux, to the extent that it even exceeded the flux from HA alone.

**Table 1:** The trend of permeate flux based on inorganic–organic interactions.

Organic Matter	Membrane Type	Organic Matter Concentration	Inorganic Matter		Filtration Time	Variation of Normalized Flux Compared with Organic Matter Fouling Only	The Trend of Permeate Flux
			Species	Concentration			
HA	UF	10 mg/L [68]	Al^3+^	0.3 mM	7.5 min	+10.21%	increase
		50 mg/L [72]	Mg^2+^	0.025 mM	60 min	−2.66%	/
			Ca^2+^			−9.37%	
			Fe^3+^			−9.03%	
		10 mg/L DOC [71]	Ca^2+^	1 mM	120 min	−43.49%	increase
				10 mM		−16.27%	
				100 mM		+8.30%	
		20 mg/L [70]	Ca^2+^	0.02 mM	5 min	−34.47%	decrease
				0.2 mM		−49.07%	
			Mg^2+^	0.02 mM		−27.33%	decrease
				0.2 mM		−44.10%	
			Al^3+^	0.02 mM		−7.55%	increase
				0.2 mM		+4.40%	
			Fe^3+^	0.02 mM		−30.19%	decrease
				0.2 mM		−21.38%	
	RO	10 mg/L [73]	Ca^2+^	1 mM	45 h	−24.12%	decrease
				3 mM		−30.35%	
				5 mM		−35.77%	
		50 mg/L [16]	Si	6 mM	10 h	−38.29%	/
Polysaccharides	UF	50 mg/L SA MM-block	Ca^2+^ [12,74]	1 mM	48 h	−53.10%	decrease
				2 mM		−63.20%	
		50 mg/L SA GG-block		1 mM	24 h	−80.50%	decrease
				2 mM		−83.86%	
		50 mg/L SA MG-block		1 mM	72 h	−10.31%	decrease
				2 mM		−12.67%	
		50 mg/L SA [45]	Ca^2+^	0.13 mM	200 min	−39.12%	decrease
				0.25 mM		−42.36%	
				0.50 mM		−44.51%	
				0.75 mM		−45.17%	
				1.00 mM		−46.41%	
				1.5 mM		−42.36%	increase
				5.00 mM		−41.11%	
				10.00 mM		−23.31%	
			Mg^2+^	0.13 mM		−28.11%	decrease
				0.25 mM		−31.86%	
				0.50 mM		−33.37%	
				0.75 mM		−36.71%	
				1.00 mM		−39.22%	
				1.5 mM		−46.21%	
				5.00 mM		−49.37%	
				10.00 mM		−51.73%	
	RO	10 mg/L Gellan gum	Ca^2+^ [38]	1 mM	45 h	−34.31%	decrease
				3 mM		−50.59%	
		10 mg/L SA		1 mM		−46.61%	decrease
				3 mM		−47.31%	
			Si [46]	2 mM	1000 min	−25.30%	/
BSA	UF	20 mg/L [69]	Al^3+^	0.1 mg/L	10 min	+27.33%	increase
				0.3 mg/L		+29.36%	
				0.9 mg/L		+9.22%	
				1.5 mg/L		+7.69%	
		500 ppm [52]	Fe^3+^	0.1 mM	100 min	−12.31%	/
			Ca^2+^			0	/
							/
	RO	50 mg/L [75]	Mg^2+^	1 mM	25 h	−30.51%	/
			Ca^2^			−36.86%	/
		35 mg/L [53]	Si	2.8 mM	500 mins	−46.66%	/
		300 mg/L [48]	Ca^2+^	0.5 mM	20 h	−2.17%	decrease
				1 mM		−18.66%	
				2 mM		−21.39%	

## 4. Conclusions and Perspectives

### 4.1. Conclusions

This mini review systematically analyzed our current understanding of the roles of inorganic matter in organic membrane fouling. The conclusions are:

1. Inorganic matters like Ca^2+^, Mg^2+^, Al^3+^, and silica readily interact with organic foulants like humic acids, polysaccharides, and proteins via mechanisms including ionic bonding, hydrogen bonding, coordination, and van der Waals forces. These facilitate the formation of larger aggregates that exacerbate membrane fouling, especially in the RO system.
2. Molecular modeling techniques, such as MD and DFT, are valuable tools for gaining molecular insights into membrane fouling, facilitating a clearer understanding of organic membrane fouling mechanisms.
3. Polysaccharide fouling is primarily governed by the formation of TEP, which is induced by the bridging of inorganic ions between polysaccharide chains.
4. Inorganic coagulants, such as aluminum (Al) and iron (Fe) salts, are capable of alleviating UF organic membrane fouling by forming loose and porous fouling layers through complexation with organic matter.

### 4.2. Perspectives

At present, membrane research has mainly focused on the interaction between a single organic substance and a single inorganic substance. In the future, molecular simulation calculations should be used to study the membrane fouling behavior caused by interactions between multiple types of organic and inorganic substances, which is more in line with practical applications. In addition, based on the enhancing effect of inorganic ions on organic membrane pollution, we recommend developing reagents similar to heavy metal catchers to precipitate complex inorganic ions from the influent, thereby alleviating membrane pollution.

## Data Availability

The data presented in this study are available upon request from the corresponding author.
